# Identification of a biomarker panel for improvement of prostate cancer diagnosis by volatile metabolic profiling of urine

**DOI:** 10.1038/s41416-019-0585-4

**Published:** 2019-10-07

**Authors:** Ana Rita Lima, Joana Pinto, Ana Isabel Azevedo, Daniela Barros-Silva, Carmen Jerónimo, Rui Henrique, Maria de Lourdes Bastos, Paula Guedes de Pinho, Márcia Carvalho

**Affiliations:** 1 UCIBIO/REQUIMTE, Department of Biological Sciences, Laboratory of Toxicology, Faculty of Pharmacy, University of Porto, Porto, Portugal; 2 Cancer Biology & Epigenetics Group, Research Center (CI-IPOP) Portuguese Oncology Institute of Porto (IPO Porto), Porto, Portugal; 3 Department of Pathology and Molecular Immunology-Biomedical Sciences Institute (ICBAS), University of Porto, Porto, Portugal; 4 Department of Pathology, Portuguese Oncology Institute of Porto (IPO Porto), Porto, Portugal; 5 UFP Energy, Environment and Health Research Unit (FP-ENAS), University Fernando Pessoa, Porto, Portugal

**Keywords:** Diagnostic markers, Cancer metabolism

## Abstract

**Background:**

The lack of sensitive and specific biomarkers for the early detection of prostate cancer (PCa) is a major hurdle to improve patient management.

**Methods:**

A metabolomics approach based on GC-MS was used to investigate the performance of volatile organic compounds (VOCs) in general and, more specifically, volatile carbonyl compounds (VCCs) present in urine as potential markers for PCa detection.

**Results:**

Results showed that PCa patients (*n* = 40) can be differentiated from cancer-free subjects (*n* = 42) based on their urinary volatile profile in both VOCs and VCCs models, unveiling significant differences in the levels of several metabolites. The models constructed were further validated using an external validation set (*n* = 18 PCa and *n* = 18 controls) to evaluate sensitivity, specificity and accuracy of the urinary volatile profile to discriminate PCa from controls. The VOCs model disclosed 78% sensitivity, 94% specificity and 86% accuracy, whereas the VCCs model achieved the same sensitivity, a specificity of 100% and an accuracy of 89%. Our findings unveil a panel of 6 volatile compounds significantly altered in PCa patients’ urine samples that was able to identify PCa, with a sensitivity of 89%, specificity of 83%, and accuracy of 86%.

**Conclusions:**

It is disclosed a biomarker panel with potential to be used as a non-invasive diagnostic tool for PCa.

## Background

Prostate cancer (PCa) ranks second in cancer incidence and fifth in mortality among men worldwide.^1^ Diagnostic strategies currently available for patients with PCa rely on prostate biopsy (PB), which is an invasive, unpleasant and potentially harmful procedure, potentially missing clinically significant cancers due to tumour heterogeneity.^2^ Prostate cancer detection based on serum PSA with a cut-off of 4.0 ng/ml has limited sensitivity (of 20.5%) and specificity (ranging from 51 to 91%),^3,4^ and inability to differentiate aggressive from indolent PCa,^4^ leading to false negatives, to overdiagnosis and consequent overtreatment.^5^ The free/total serum PSA ratio (fPSA/tPSA) has been proposed as an alternative. However, it displays the opposite performance, with high sensitivity but low specificity.^3^ Globally, this entails the performance of a large number of prostate biopsies, a significant proportion of which is deemed unnecessary. Thus, the free/total PSA ratio is not usually employed for risk-stratification of prostate cancer, but only in selected cases. The reported values for the sensitivity and specificity of this biomarker are very inconsistent among different studies, nevertheless a recent meta-analysis concluded that this biomarker shows a sensitivity of 70% and a specificity of 58%.^6^ Thus, intense efforts have been devoted for development of PCa molecular biomarkers, some of which have already obtained FDA approval, like prostate cancer antigen 3 (PCA3)^7^ or circulating tumour cells (CTs).^7^ Notwithstanding, these biomarkers also have important limitations, such as the definition of a cut-off value (e.g., PCA3)^7^ and low abundance at early stages (e.g., CTs).^7^ Thus, discovery and validation of novel PCa biomarkers with improved sensitivity, non-invasive and able to detect early-stage disease (when PCa is potentially curable) remains an important research aim.

Metabolomics emerged as one of the most promising approaches for discovery of new disease biomarkers as pathological conditions cause disruption of metabolic processes and consequently change the production, use and levels of many metabolites, resulting in a characteristic “metabolic signature” that can be captured through metabolic profiling. Analysis of the volatile part of the metabolome, i.e. the low molecular weight volatile organic compounds (VOCs) present in the headspace (gas phase) of clinical samples (e.g., biofluids as urine), is a promising new screening tool for several cancers, including PCa.^8–10^ VOCs are end products of cellular activities and alterations in VOCs profile may reflect modifications in gene activation, gene expression, proteins and activity of enzymes involved in metabolic pathways. These volatile molecules endow biological samples with distinct odours which may even be detected by animals with highly sensitive olfactory capabilities, such as dogs,^11,12^ or sophisticated analytical instrumental techniques, such as gas chromatography-mass spectrometry (GC-MS) combined with multivariate statistical analysis (MVA).^8–10^ In this regard, Smith et al.^8^ studied the urine metabolomics of 13 PCa patients and 24 controls using GC-MS, disclosing 91 VOCs and unveiling significant differences between PCa and controls in 21 VOCs. However, this study has important limitations namely a small sample size and lack of external validation.^8^ Khalid et al. performed the GC-MS volatile profiling of urine from PCa patients using a larger number of samples (*n* = 59 PCa and *n* = 43 controls). Overall, 196 VOCs were identified from which four (2,6-dimethy-7-octen-2-ol, pentanal, 3-octanone, and 2-octanone) were found to be statistically different between PCa and control samples.^9^ More recently, Jimenez-Pacheco et al. performed a similar study using 29 PCa urine samples that were compared with 21 samples from patients with benign prostatic hyperplasia (BPH). In this study, 57 VOCs were identified, but only nine significantly differed between the two groups, highlighting furan and p-xylene as potential PCa biomarkers.^10^ Interestingly, 2-octanone^8,9^ and 2,6-dimethy-7-octen-2-ol^9,10^ were pointed as urinary PCa biomarkers in more than one study. Taken together, these studies provide convincing evidence that volatiles emanating from urine are potential biomarkers for PCa detection. Recently, the feasibility and potential of volatile signature for diagnosing PCa led to the development of chemical system sensors (so-called “electronic nose” or “e-nose”).^13,14^ “E-noses” are designed to mimic the mammalian olfactory system and provide a global characterisation of the odorous mixtures.^15^ Remarkably, the application of the “e-nose” technology to discriminate the odour of urine from patients with PCa from controls provided better diagnostic performance than serum PSA.^13,14^

Herein, we aimed to obtain a more comprehensive metabolomic profiling of volatile metabolites in urine from PCa patients, using a metabolomics approach based on headspace solid-phase microextraction coupled with GC-MS (HS-SPME/GC-MS). Two different sample preparation strategies were considered: (i) direct analysis for VOCs detection and (ii) derivatisation with O-(2,3,4,5,6-pentafluorobenzyl)hydroxylamine (PFBHA), prior to HS-SPME/GC-MS analysis, to enhance the sensitive detection of volatile carbonyl compounds (VCCs). An external validation set was then used to validate a panel of discriminant volatile compounds with clinical potential for PCa diagnosis. To the best of our knowledge, this is the first time that VCCs are investigated as urinary PCa biomarkers and that a volatile biomarker panel for PCa is validated using an external set of samples.

## Methods

### Chemicals

All chemicals used were of analytical grade. Benzaldehyde (≥99.5%), 2-butanone (≥99%), (E)-2-butenal (≥99%), cyclohexanone (≥99%), 2-decanone (≥98%), (E)-2-decenal (≥92%), 2,5-dimethylbenzaldhyde (≥99%), 3,4-dimethylcyclohex-3-ene-1-carbaldehyde (≥97%), 2,6-dimethyl-6-hepten-2-ol (≥96%), 3,7-dimethylocta-1,6-dien-3-ol (≥95%), 4-fluorobenzaldehyde (≥98%), 2-furfural (≥99%), heptanal (≥92%), 4-heptanone (≥97%), hexadecane (≥99%), (E,E)-2,4-hexadienal (≥95%), hexanal (≥98%), 2-hexanone (≥98%), 2-hydroxy-2-methyl-1-phenylpropan-1-one (≥97%), 2-methylbutanal (≥90%), 3-methylbutanal (≥97%), 2-methylcyclopentan-1-one (≥97%), 5-methyl-2-furfural (≥99%), methylglyoxal (40% aqueous solution), 5-methylheptan-2-one (≥95%), 2-methylpropanal (≥98%), 5-methyl-2-(propan-2-yl) cyclohexyl acetate (≥98%), nonanal (≥95%), 2-nonanone (≥97%), (E)-2-nonenal (≥93%), octanal (≥98%), 2-octanone (≥98%), pentanal (≥97%), (E)-2-pentenal (≥95%), 3-penten-2-one (≥70%), 3-phenylpropionaldehyde (≥95%), PFBHA (≥98%), phenylacetaldehyde (≥90%), propanal (≥97%), terpinen-4-ol (≥95%), 2,6,6,10-tetramethyl-1-oxaspiro[4.5]dec-9-ene (≥90%), and 3,7,7-trimethylbicyclo[4.1.0] hept-3-ene (≥97%) were purchased from Sigma–Aldrich (Madrid, Spain). Butanal (≥99%) and glyoxal (≥95%) were purchased from Fluka (Madrid, Spain) and 4-hydroxy-2-nonenal (≥98%) was purchased from Cayman Chemical (USA). Sodium chloride was obtained from VWR (Leuven, Belgium).

### Subjects

Early morning urine samples without fasting were collected from PCa patients and controls at the Portuguese Oncology Institute of Porto (IPO Porto) and frozen at −80 °C until analysis. The study protocol was approved by the local Ethics Committee and all subjects provided their signed informed consent prior to enrolment.

A cohort of 118 men were included in this study: 58 PCa patients (age 52–77 years, mean 63) and 60 cancer-free control subjects (age 56–66 years, mean 59). Both PCa and control groups were randomly divided into two sets: (1) training (*n* = 40 PCa and *n* = 42 controls for VOCs; *n* = 40 PCa and *n* = 40 controls for VCCs) and (2) external validation (*n* = 18 PCa and *n* = 18 controls for VOCs and VCCs). Control group consisted of subjects with age-related comorbidities such as hypertension, diabetes, lipid disorders and BPH, but without cancer. Detailed information on Gleason score and some important biochemical and clinical parameters of PCa patients and control subjects is provided in Table 1.

**Table 1:** Demographic and clinical data of the PCa patients and cancer-free controls included in the training and validation sets

Characteristics	Prostate cancer				Control			
	Training set VOCs	External set VOCs	Training set VCCs	External set VCCs	Training set VOCs	External set VOCs	Training set VCCs	External set VCCs
Number of subjects	40	18	40	18	42	18	40	18
Mean Age ± SD (years)	64.4 ± 6.4	61.8 ± 5.2	63.7 ± 6.5	63.4 ± 5.3	59.3 ± 3.0	59.6 ± 2.62	59.3 ± 2.8	59.8 ± 2.7
PSA (ng/mL), *n* (%)								
<4	3 (7.5%)	1 (5.6%)	–	4 (22.2%)	–	–	–	–
4–10	24 (60%)	13 (72.2%)	28 (70%)	9 (50%)	–	–	–	–
>10	13 (32.5%)	4 (22.2%)	12 (30%)	5 (27.8%)	–	–	–	–
Gleason score, *n* (%)								
≤6	6 (15%)	3 (16.7%)	8 (20%)	1 (5.6%)	–	–	–	–
=7	25 (62.5%)	12 (66.7%)	24 (60%)	13 (72.2%)	–	–	–	–
≥8	9 (22.5%)	3 (16.7%)	8 (20%)	4 (22.2%)	–	–	–	–
Clinical stage, *n* (%)								
I	3 (7.5%)	3 (16.7%)	4 (10%)	2 (11.1%)	–	–	–	–
II	–	2 (11.1%)	2 (4%)	–	–	–	–	–
IIA	7 (17.5%)	4 (22.2%)	9 (22.5%)	2 (11.1%)	–	–	–	–
IIB	15 (37.5%)	2 (11.1%)	11 (27.5%)	6 (33.3%)	–	–	–	–
III	13 (32.5%)	5 (27.8%)	10 (25%)	8 (44.4%)	–	–	–	–
IV	2 (5%)	2 (11.1%)	4 (10%)	–	–	–	–	–
Alcoholism, *n* (%)	7 (17.5%)	4 (22.2%)	9 (22.5%)	2 (11.1%)	3 (7.1%)	–	2 (5%)	1 (5.6%)
Smoking, *n* (%)	2 (5%)	–	2 (5%)	–	5 (11.9%)	2 (11.1%)	6 (15%)	1 (5.6%)
Obesity, *n* (%)	6 (15%)	4 (22.2%)	7 (17.5%)	3 (16.7%)	7 (16.7%)	3 (16.7%)	7 (17.5%)	2 (11.1%)
Cardiac condition, *n* (%)	5 (12.5%)	6 (33.3%)	7 (17.5%)	4 (22.2%)	–	1 (5.6%)	–	1 (5.6%)
AH, *n* (%)	21 (52.5%)	8 (44.4%)	19 (47.5%)	10 (55.6%)	14 (33.3%)	9 (50%)	20 (50%)	3 (16.7%)
Dyslipidemia, *n* (%)	16 (40%)	8 (44.4%)	14 (35%)	10 (55.6%)	16 (38.1%)	9 (50%)	16 (40%)	8 (44.4%)
Diabetes, *n* (%)	9 (22.5%)	3 (16.7%)	8 (20%)	4 (22.2%)	6 (14.3%)	1 (5.6%)	5 (12.5%)	1 (5.6%)
HTG, *n* (%)	2 (5%)	–	1 (2.5%)	1 (5.6%)	1 (2.4%)	–	–	1 (5.6%)
HC, *n* (%)	3 (7.5%)	–	1 (2.5%)	2 (11.1%)	4 (9.5%)	1 (5.6%)	3 (7.5%)	2 (11.1%)
BPH, *n* (%)	–	–	–	–	13 (31%)	4 (22.2%)	11 (27.5%)	4 (22.2%)
Prostatitis, *n* (%)	–	–	–	–	1 (2.4%)	1 (5.6%)	2 (5%)	–

*AH* arterial hypertension, *BPH* benign prostatic hyperplasia, *HC* hypercholesteremia, *HT*G hypertriglyceridemia

### Sample preparation and metabolites extraction

Urine samples were thawed at 4 °C. For VOCs analysis, 1 mL of sample was placed in a 10 mL glass vial with 20 µL of internal standard (IS) (10 μg/mL 4-fluorobenzaldehyde in ultrapure water) and NaCl (0.27 g). To optimise the extraction conditions, a central composite design (CCD) was performed (data not shown). The optimal extraction conditions, using divinylbenzene/carboxen/polydimethylsiloxane (DVB/CAR/ PDMS) fiber coating, were 11 min of incubation and 30 min of extraction at 44 °C under continuous stirring (250 rpm).

For VCCs analysis, 250 µL of urine were placed in a 10 mL glass vial with 5 µL of IS (10 μg/mL 4-fluorobenzaldehyde in ultrapure water) and 7.5 µL of the derivatizing agent PFBHA (40 g/L in ultrapure water). Extraction was performed according to the conditions previously optimised in our lab^16^ using a CombiPAL automatic autosampler (Varian, Palo Alto, CA) and a polydimethylsiloxane/divinylbenzene (PDMS/DVB) fiber coating. Briefly, urine samples were incubated at 62 °C during 6 min, followed by extraction of volatiles at the same temperature during 51 min, under continuous stirring (250 rpm). After extraction, the fiber was inserted into the GC system for thermal desorption of the analytes at 250 °C during 5 min.

In both approaches, all samples were randomly injected, with the quality control (QCs) samples being injected at the same conditions on every eight samples. QCs were prepared as aliquots of a pool of all urine samples (PCa and controls) considered in this study.

### GC-MS analysis

A Scion 436-gas chromatograph coupled to a Bruker single quadrupole (SQ) equipped with a Scion SQ ion trap mass detector and a Bruker Daltonics MS workstation software version 6.8, with a Rxi-5Sil MS (30 m × 0.25 mm × 0.25 μm) column from RESTEK were used. Briefly, the carrier gas was helium C-60 (Gasin, Portugal) (flow rate 1 mL/min) and the injector port was heated at 230 °C. The oven temperature was fixed at 40 °C for 1 min, increasing to 250 °C (rate 5 °C/min), held for 5 min, followed by increasing to 300 °C (rate 5 °C/min) and held for 1 min. The temperatures of transfer line, manifold and trap were 280 °C, 50 °C and 180 °C, respectively. The emission current was 50 μA and the electron multiplier was set in relative mode to an auto tune procedure. All mass spectra were acquired in the electron impact mode (270 °C). The analysis was performed in full scan mode and the mass range used was 40–350 *m/z*, with a scan rate of 6 scan/s.^17^

To analyse VCCs, a 436-GC model (Bruker Daltonics) coupled to an EVOQ triple quadrupole mass spectrometer (Bruker Daltonics) and a Bruker MS workstation software version 8.2 were used. The chromatographic separation was accomplished using a fused silica capillary column (Rxi-5Sil MS; 30 m × 0.25 mm × 0.25 μm; Restek Corporation, U.S., Bellefonte, Pennsylvania) and high purity helium C-60 (Gasin, Portugal) as carrier gas (flow rate 1 mL/min). The oven temperature was held at 40 °C for 1 min, increasing to 250 °C (rate 5 °C/min), held for 5 min, finally increasing to 300 °C (rate 20 °C/min). The temperature of transfer line and manifold were 260 °C and 40 °C, respectively. The emission current was 50 μA and the electron multiplier was set in relative mode to an auto tune procedure. All mass spectra were acquired in the electron impact mode (270 °C). Data acquisition was performed in full scan mode and a 50–600 *m/z* mass range was used.^16^

The metabolite identification was accomplished by comparison of the MS spectra with standards (whenever available), the National Institute of Standards and Technology (NIST 14) database spectral library, and comparison of the experimental and theory (literature) Kovats index.

### Data pre-processing

Before statistical analysis, the data was pre-processed using MZmine 2,^18^ including baseline correction, peak detection, chromatogram deconvolution and alignment. The parameters used for pre-processing of VOCs data were: RT range 2.0–29.0 min, *m/z* range 50–400, MS data noise level 1.0 × 10^5^, *m/z* tolerance 0.2, chromatogram baseline level 1.0 × 10^2^ and peak duration range 0.06–0.70 min; whereas for VCCs were: RT range 6.5–38.0 min, *m/z* range 50–600, MS data noise level 5.0 × 10^5^, *m/z* tolerance 0.2, chromatogram baseline level 1.0 × 10^4^ and peak duration range 0.06–0.70 min. In both approaches, all RT-*m/z* pairs with a relative standard deviation greater than 30% in QCs, as well as RT-*m/z* pairs identified as contaminants (from column, fiber, among others), were manually removed from the matrix. The obtained data were normalised by the total area of the chromatograms and the final matrix was scaled to pareto. Furthermore, to reduce the variation from uncontrolled confounding factors and simplify the data, a variable selection method based in a univariate test,^19^ namely *t*-test, was performed using MetaboAnalyst.^20^ Consequently, all variables with *p-value* *>* 0.05 were removed from the matrix.

### Statistical analysis

The statistical analysis strategy used for VOCs and VCCs data was similar and included multivariate and univariate statistical tests. From all available samples, 70% were used for the training set and 30% were randomly selected for the external set. MVA was performed using the training set and included principal component analysis (PCA) and partial least squares discriminant analysis (PLS-DA) in SIMCA-P 15 (Umetrics, Sweden). The robustness of the PLS-DA models was confirmed through 7-fold cross validation and permutation test (200 random permutations of Y-observations, 2 components) (SIMCA-P 15, Umetrics, Sweden). To test the validity of the created models, an internal (training set) and external (external set) validation was performed. For internal and external validations, receiver operating characteristic curves (ROC), area under the curve (AUC), sensitivity, and specificity were computed (MetaboAnalyst)^20^ for both PLS-DA models (VOCs and VCCs). The samples of the external set were classified as cancer or controls, taking into consideration the PLS-DA models obtained using the training sets and the sensitivity, specificity and accuracy of both PLS-DA models (VOCs and VCCs) were computed.^21^

After MVA, all metabolites with VIP (Variable Importance to the Projection) greater than one were subjected to univariate analysis (GraphPad Prism 6, USA), including a normality test (Shapiro-Wilk test) followed by unpaired Student’s *t*-test with Welch correction test, for normal distribution, or unpaired Mann–Whitney *U*-test, for non-normal distribution. Percentage of variation, uncertainty of the percentage of variation, and effect size and the standard error were also determined.^22^ For all significantly altered metabolites (*p*-value < 0.05 and effect size higher than the standard error), receiver operating characteristic curves (ROC), area under the curve (AUC), sensitivity, and specificity were also computed (MetaboAnalyst).^20^ Bonferroni correction was used to adjust *p*-values in multiple comparisons.^23^ Multivariate ROC exploratory analysis (Metaboanalyst)^20^ was used to define a small panel of discriminant metabolites with high accuracy for prostate cancer detection, envisaging a possible translation into clinics using an “e-nose”. The PLS-DA algorithm was used to evaluate the importance of each discriminant metabolite based on VIP scores through repeated random sub-sampling cross validation. The top important metabolites were used to build a PLS-DA model which was validated through ROC analysis using the training and external sets.

To better understand the biological relevance of the significantly altered VOCs and VCCs, a metabolic pathway analysis using the MetPa tool was performed in Metaboanalyst.^20^ Finally, to search for possible correlations between the metabolites significantly altered in PCa, Spearman’s rank correlation coefficient was computed for the set of identified and putatively annotated statistically significant compounds and represented in a heatmap, using R software (version 3.5.1).^24^ Spearman’s rank correlation coefficient was also computed between age and the set of metabolites found altered in PCa compared to controls.

## Results

### Urinary volatile profile of PCa patients vs. controls

In this study, a HS-SPME/GC-MS method was employed to evaluate differences in the urinary volatile profile of PCa patients compared with controls. To accomplish a more comprehensive evaluation of the urinary volatilome, we used two different sample preparation techniques which enabled the identification of 122 VOCs and 148 VCCs (seven common compounds were found).

**Fig. 1:**
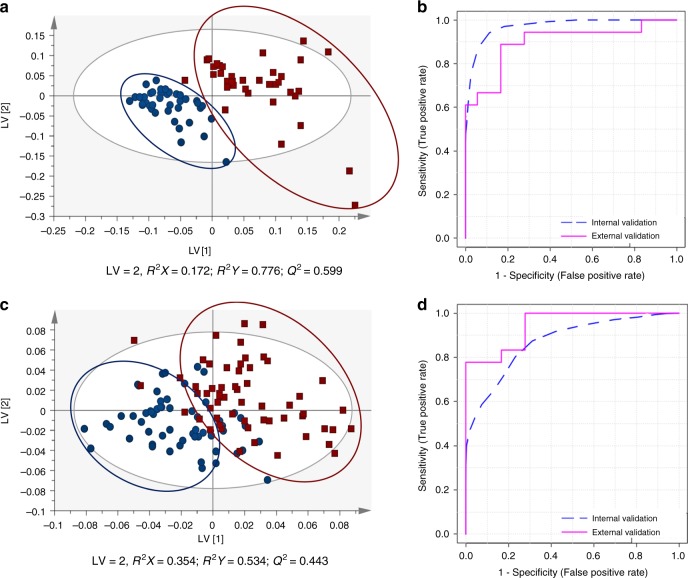
**a** PLS-DA scores scatter plot (Pareto scaling; 2 components) obtained for VOCs training model of PCa patients (*n* = 40, squares) vs. cancer-free controls (*n* = 42, circles), after variable selection; **b** Assessment of the diagnostic performance of the PLS-DA model obtained for VOCs using the training set (AUC = 0.975; sensitivity = 92%; specificity = 100%) and the external set (AUC = 0.898; sensitivity = 78%; specificity = 94%) through ROC analysis; **c** PLS-DA scores scatter plot (Pareto scaling; 2 components) obtained for VCCs training model of PCa patients (*n* = 40, squares) vs. cancer-free controls (*n* = 40, circles), after variable selection; **d** Assessment of the diagnostic performance of the PLS-DA model obtained for VCCs using the training set (AUC = 0.878; sensitivity = 71%; specificity = 97%) and the external set (AUC = 0.944; sensitivity = 78%; specificity = 100%) through ROC analysis

MVA was used to evaluate the reproducibility of both analytical strategies and the discriminant capability of the PLS-DA models created using the training set. The QC samples were closely clustered in the PCA scores scatter plot (Fig. S1), which confirmed the analytical reproducibility of both methods. For construction of the PLS-DA models, a variable selection method was performed (VOCs: 3232 variables x 82 samples; VCCs: 246 variables x 80 samples) to improve the prediction power. In Fig. 1, the discriminant capability of the PLS-DA models, after variable selection, is clearly observed (VOCs model: LV = 2; R^2^X = 0.172; R^2^Y = 0.776; Q^2^ = 0.599; VCCs model: LV = 2; R^2^X = 0.354; R^2^Y = 0.534; Q^2^ = 0.443). Model robustness was also confirmed through permutation testing (Fig. S2). In the internal validation,  VOCs PLS-DA model showed an AUC of 0.975, a sensitivity of 92% and specificity of 100% and the VCCs model unveiled an AUC of 0.878, a sensitivity of 71% and specificity of 91% (Fig. 1).

Furthermore, an external validation set was used to confirm the validity of the training models. For VOCs and VCCs, among 18 PCa samples, 14 were accurately classified and four were poorly classified. On the other hand, 17 control samples were accurately classified and only one was poorly classified for VOCs, whereas all 18 control samples were correctly classified for VCCs (Table S3). Thus, taking into consideration these results, a sensitivity of 78%, a specificity of 94% and an accuracy of 86% was obtained for VOCs, whereas VCCs disclosed equal sensitivity, a specificity of 100% and an accuracy of 89%. For VOCs, from a total of 64 metabolites with VIP > 1, 31 were found significantly different between the two groups (PCa vs. control). The discriminant VOCs included three aldehydes, six ketones, two alcohols, two monoterpene alcohols, one alkene, one cycloalkane, two terpenes, among others, and 11 unidentified compounds (Table 2). Regarding VCCs analysis, 21 metabolites showed VIP > 1 and 12 significantly differed between PCa and control groups. The discriminant VCCs included two alpha-ketoaldehydes, one alkanal, one alkenal, two aromatic aldehydes, three ketones, one alkane and two unidentified compounds (Table 3). The chromatographic characteristics considered for identification of VOCs and VCCs are displayed in Tables S1 and S2, respectively. AUC values were superior to 0.6 for all statistically significantly altered metabolites (Tables 2 and 3). The sensitivity and specificity of the individual metabolites was also determined and, despite the lower individual sensitivity and specificity found for the majority of the metabolites when compared to the one obtained for the models (Fig. 1 and Table S3), all metabolites disclosed sensitivity and specificity greater than 50 and 70%, respectively (Tables 2 and 3).

**Table 2:** List of VOCs significantly altered in PCa group compared to controls

Chemical name (IUPAC) or common name	*p*-value	Variation ± uncertainty (%)	Effect size ± ES_SE_	AUC	Spec.	Sens.	HMDB^29^	Matrices	Potential biochemical pathway
Aldehydes									
Hexanal ^L1^	0.0313	↓ 14.62 ± 6.77	↓ 0.53 ± 0.45	0.641	0.76	0.51	HMDB0005994	Blood; Cerebrospinal fluid; Feces; Saliva; Urine^29^	Steroid hormone biosynthesis^20^
3,4-Dimethylcyclohex-3-ene-1-carbaldehyde ^L1^	0.0004^B^	↓ 24.09 ± 8.68	↓ 0.71 ± 0.46	0.730	0.84	0.61	NA	–	–
2,5-Dimethylbenzaldehyde ^L1^	<0.0001^B^	↑ 49.36 ± 9.90	↑ 0.91 ± 0.47	0.786	0.87	0.64	HMDB0032014	–	Alcohols and fatty acids metabolism^40,48^
Ketones									
Hexan-2-one ^L1^(2-Hexanone)	0.0194	↓ 23.42 ± 10.77	↓ 0.56 ± 0.45	0.656	0.77	0.53	HMDB0005842	Urine; Feces^29^	Fatty acid metabolism^41^
2-Methylcyclopentan-1-one ^L1^	0.0129	↓ 31.26 ± 12.85	↓ 0.65 ± 0.46	0.662	0.78	0.55	NA	–	Fatty acid metabolism^41^
4-Methylhexan-3-one ^L2^	0.0022	↓ 16.49 ± 6.17	↓ 0.66 ± 0.46	0.701	0.82	0.59	NA	–	Fatty acid metabolism^41^
5-Methylheptan-2-one ^L1^	0.0073	↓ 21.40 ± 11.34	↓ 0.48 ± 0.45	0.677	0.80	0.51	NA	Cell lines^40^	Fatty acid metabolism^41^
4,6-Dimethylheptan-2-one ^L2^	0.0174	↓ 17.04 ± 6.67	↓ 0.63 ± 0.46	0.658	0.76	0.55	NA	–	Fatty acid metabolism^41^
2-Hydroxy-2-methyl-1-phenylpropan-1-one ^L1^	0.0123	↓ 11.90 ± 4.03	↓ 0.71 ± 0.46	0.662	0.78	0.55	NA	–	–
Alcohols									
2,6-Dimethyl-6-hepten-2-ol ^L1^	0.0002^B^	↓ 36.42 ± 12.89	↓ 0.78 ± 0.46	0.748	0.84	0.63	NA	–	Lipid metabolism^40^
1-Methyl-4-propan-2-ylcyclohex-2-en-1-ol ^L2^	0.0026	↓ 13.49 ± 5.75	↓ 0.57 ± 0.45	0.698	0.81	0.57	NA	–	Lipid metabolism^40^
Monoterpene alcohols									
3,7-Dimethylocta-1,6-dien-3-ol (Linalool)^L1^	0.0355	↓ 28.00 ± 13.53	↓ 0.55 ± 0.45	0.635	0.75	0.51	HMDB0036100	Feces^29^	Lipid metabolism^29^
4-Methyl-1-propan-2-ylcyclohex-3-en-1-ol ^L1^ (Terpinen-4-ol)	<0.0001^B^	↓ 28.84 ± 8.35	↓ 0.91 ± 0.47	0.766	0.87	0.65	HMDB0035833	Feces; Cell lines^29,40^	Lipid metabolism^29^
Alkenes									
4-Methyldec-1-ene ^L2^	0.0321	↓ 18.31 ± 9.75	↓ 0.47 ± 0.45	0.635	0.75	0.52	NA	–	
Cycloalkenes									
2,2,7,7-Tetramethyltricyclo[6.2.1.0¹,^6^]undeca-3,5,9-triene (4,5,9,10-dehydroisolongifolene) ^L2^	0.0379	↓ 15.98 ± 8.18	↓ 0.48 ± .045	0.634	0.76	0.50	HMDB0059829	Saliva^29^	Steroid metabolism^49^
Terpenes									
3,7,7-Trimethylbicyclo[4.1.0] hept-3-ene (3-Carene) ^L1^	0.0108	↓ 16.72 ± 6.46	↓ 0.64 ± 0.46	0.672	0.78	0.54	HMDB0035619	Feces^29^	Lipid metabolism^29^
3-Methyl-6-(propan-2-ylidene)cyclohex-1-ene (Isoterpinolene) ^L2^	0.0062	↓ 18.79 ± 8.17	↓ 0.58 ± 0.45	0.676	0.79	0.56	HMDB0061938	Saliva^29^	Lipid metabolism^29^
Others									
2,2,2,8a-Tetramethyl-3,4,4a,5,6,8a-hexahydro-2H-chromene (Dihydroedulan IA) ^L2^	0.0251	↓ 12.58 ± 5.46	↓ 0.56 ± 0.45	0.629	0.77	0.52	NA	–	–
5-Methyl-2-(propan-2-yl)cyclohexyl acetate ^L1^ (Menthyl acetate)	0.0139	↓ 12.89 ± 5.09	↓ 0.61 ± 0.45	0.662	0.77	0.54	HMDB0041264	–	Lipid metabolism^29^
2,6,6,10-Tetramethyl-1-oxaspiro[4.5]dec-9-ene (Theaspirane) ^L1^	0.0096	↓ 13.36 ± 5.23	↓ 0.62 ± 0.45	0.668	0.78	0.55	HMDB0036823	Urine^29^	Energetic metabolism; cell signalling; membrane stabilisation^29^
Unidentified VOCs									
Unknown 1 ^L4^	0.0137	↓ 12.70 ± 6.38	↓ 0.48 ± 0.45	0.661	0.77	0.53	NA	–	–
Unknown 2 ^L4^	<0.0001^B^	↓ 49.99 ± 17.13	↓ 0.88 ± 0.47	0.822	0.92	0.73	NA	–	–
Unknown 3 ^L4^	0.0006^B^	↓ 23.54 ± 8.13	↓ 0.74 ± 0.46	0.727	0.84	0.62	NA	–	–
Unknown 4 ^L4^	<0.0001^B^	↓ 55.31 ± 13.34	↓ 1.30 ± 0.49	0.883	0.95	0.80	NA	–	–
Unknown 5 ^L4^	0.0031	↓ 14.50 ± 6.14	↓ 0.58 ± 0.45	0.695	0.81	0.58	NA	–	–
Unknown 6 ^L4^	<0.0001^B^	↓ 71.24 ± 10.61	↓ 1.12 ± 0.48	0.768	0.86	0.66	NA	–	–
Unknown 7 ^L4^	0.0093	↓ 15.01 ± 6.08	↓ 0.61 ± 0.45	0.665	0.77	0.54	NA	–	–
Unknown 8 ^L4^	0.0056	↓ 16.23 ± 7.55	↓ 0.53 ± 0.45	0.684	0.81	0.60	NA	–	–
Unknown 9 ^L4^	<0.0001^B^	↓ 16.12 ± 6.57	↓ 0.60 ± 0.45	0.808	0.90	0.70	NA	–	–
Unknown 10 ^L4^	0.0205	↓ 16.39 ± 7.42	↓ 0.55 ± 0.45	0.659	0.76	0.54	NA	–	–
Unknown 11 ^L4^	0.0043	↓ 21.05 ± 7.98	↓ 0.69 ± 0.46	0.677	0.78	0.55	NA	–	–

The statistical significance (*p*-values), percentage of variation, effect size (ES), standard error (ES_SE_), AUC, specificity (spec.) and sensitivity (sens.) are represented for each VOC, as well as the HMDB (human metabolome database) code (when available), the matrices where the compound was previously found and the potential biochemical pathways where the compound participates

*NA* not available

^L1^Identified metabolites (GC-MS analysis of the metabolite of interest and a chemical reference standard of suspected structural equivalence, with all analyses performed under identical analytical conditions within the same laboratory)^54^

^L2^Putatively annotated compounds (spectral (MS) similarity with NIST database), when standards were not commercially available^54^

^L4^Unidentified^54^

^B^Alterations remaining significant after Bonferroni correction, with cut-off *p*-value of 7.69 × 10^−4^ (0.05 divided by 65 analysed VOCs)

**Table 3:** List of VCCs significantly altered in PCa group compared to controls

Chemical name (IUPAC) or common name	*p*-value	Variation ± uncertainty (%)	Effect size ± ES_SE_	AUC	Spec.	Sens.	HMDB^29^	Matrices	Potential biochemical pathway
Alpha-ketoaldehydes									
Oxaldehyde ^L1^ (Glyoxal)	0.0342	↓ 8.67 ± 4.23	↓ 0.48 ± 0.44	0.612	0.73	0.48	NA	–	Peroxidation of polyunsaturated fatty acids^50^
2-Oxopropanal ^L1^ (Methylglyoxal/ Pyruvaldehyde)	0.0101	↓ 22.35 ± 9.58	↓ 0.59 ± 0.45	0.638	0.76	0.53	HMDB01167	Urine; Blood^29^	Pyruvate metabolism; Glycine, serine and threonine metabolism^50^
Alkanals									
Decanal ^L1^	0.0210	↓ 18.28 ± 7.52	↓ 0.60 ± 0.45	0.649	0.76	0.55	HMDB0011623	Saliva; Feces; Urine; Blood^29^	Alcohols and fatty acids metabolism; amino acids and carbohydrate catabolism^40,48^
Alkenals									
But-2-enal ^L1^ (2-Butenal)	0.0040	↓ 22.64 ± 7.33	↓ 0.78 ± 0.46	0.686	0.78	0.56	HMDB0034233	Feces; Saliva^29^	Lipid peroxidation^51,52^
Alkanes									
Hexadecane ^L1^	0.0308	↑ 30.23 ± 10.86	↑ 0.54 ± 0.45	0.642	0.76	0.51	HMDB33792	Feces; Saliva^29^	NA
Ketones									
Butan-2-one ^L1^ (2-Butanone)	0.0003^B^	↑ 39.88 ± 8.81	↑ 0.84 ± 0.45	0.732	0.83	0.61	HMDB0000474	Saliva; Feces; Urine; Blood^10,29^	Fatty acid and carbohydrate metabolisms^53^
Pentan-2-one ^L1^ (2-Pentanone)	0.0356	↑ 53.36 ± 18.02	↑ 0.52 ± 0.45	0.638	0.75	0.51	HMDB34235	Saliva; Feces; Urine^29^	Fatty acid metabolism^41^
Cyclohexanone ^L1^	0.0021^B^	↑ 30.89 ± 8.65	↑ 0.69 ± 0.45	0.704	0.82	0.59	HMDB0003315	Feces^29^	Fatty acid metabolism^41^
Aromatic aldehydes									
3-Phenylpropanal ^L1^ (3-Phenylpropionaldehyde)	<0.0001^B^	↑ 38.35 ± 7.11	↑ 1.01 ± 0.47	0.757	0.85	0.65	HMDB33716	-	Alcohols and fatty acids metabolism; amino acids and carbohydrate catabolisms^40,48^
2-Phenylacetaldehyde ^L1^ (Phenylacetaldehyde)	<0.0001^B^	↑ 50.66 ± 15.08	↑ 0.60 ± 0.45	0.765	0.85	0.65	HMDB06236	Feces^29^	Phenylalanine metabolism^20^
Unidentified VCCs									
Unknown 12 ^L4^	0.0026	↑ 136.48 ± 25.12	↑ 0.72 ± 0.45	0.698	0.81	0.58	NA	–	–
Unknown 13 ^L4^	0.0126	↓ 21.71 ± 8.37	↓ 0.65 ± 0.45	0.669	0.78	0.54	NA	–	–

The statistical significance (*p*-values), percentage of variation, effect size (ES), standard error (ES_SE_), AUC, specificity (spec.) and sensitivity (sens.) are represented for each VCC, as well as the HMDB (human metabolome database) code (when available), the matrices where the compound was previously found and the potential biochemical pathways where the compound participates

*NA* not available

^L1^Identified metabolites (GC-MS analysis of the metabolite of interest and a chemical reference standard of suspected structural equivalence, with all analyses performed under identical analytical conditions within the same laboratory)^54^

^L2^Putatively annotated compounds (spectral (MS) similarity with NIST database) when standards were not commercially available^54^

^L4^Unidentified^54^

^B^Alterations remaining significant after Bonferroni correction, with cut-off *p*-value of 0.0025 (0.05 divided by 20 analysed VCCs)

Age (Table 1) significantly differed between PCa and controls in VOCs (Mann–Whitney test *p*-value = 0.0002) and VCCs (Mann–Whitney test *p*-value = 0.0022) training sets. Hence, a possible influence of age in the set of metabolites found altered in PCa compared to controls (Tables 2 and 3) was investigated through Spearman correlation, unveiling no statistically relevant correlations (|*r*| ≤ 0.36) (Table S4). In addition, the number of individuals with arterial hypertension (AH) was higher in PCa group compared to controls in the VOCs training set (Table 1). The impact of AH on urine volatile profile was evaluated in the control group (AH *n* = 14 vs. non-HA *n* = 28), revealing no predictive power (Q^2^ = −0.145) in the PLS-DA model (Fig. S3). Taking into consideration these results, no age- and AH-related changes were found in the urinary volatile signature of PCa patients.

### Definition of a multi-biomarker panel for PCa diagnosis

**Fig. 2:**
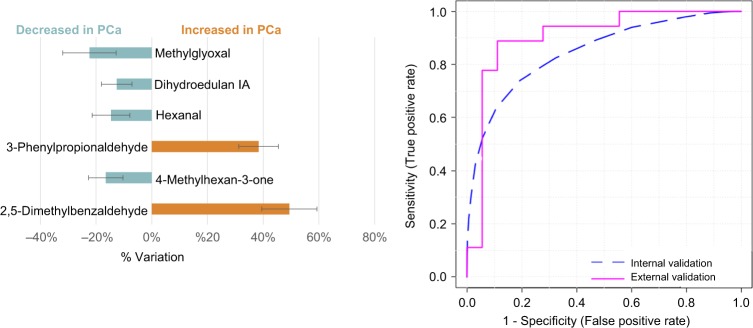
Description, % of variation and assessment of the diagnostic performance of the 6-biomarker panel using the training (AUC = 0.856; sensitivity = 72%; specificity = 96%) and the external (AUC = 0.904; sensitivity = 89%; specificity = 83%) sets through ROC analysis

The smallest panel of metabolites that best predict PCa comprised 6 metabolites, namely hexanal, 2,5-dimethylbenzaldehyde, 4-methylhexan-3-one, dihydroedulan IA, methylglyoxal and 3-phenylpropionaldehyde. This panel showed an AUC of 0.856, a sensitivity of 72%, a specificity of 96% and an accuracy of 79% taking into consideration the internal validation (Fig. 2). Regarding the external validation set, the 6-biomarker panel showed an AUC of 0.904, a sensitivity of 89%, a specificity of 83% and an accuracy of 86% (Fig. 2 and Table S5).

Although integration of volatile compounds in specific biochemical pathways is still difficult to accomplish, MetPA tool^20^ was used for identification of the most relevant metabolic pathways where the discriminant compounds are involved. The results revealed that methylglyoxal is involved in pyruvate metabolism and glycine, serine and threonine metabolism, phenylacetaldehyde in phenylalanine metabolism and hexanal in steroid hormone biosynthesis (Fig. S4).

**Fig. 3:**
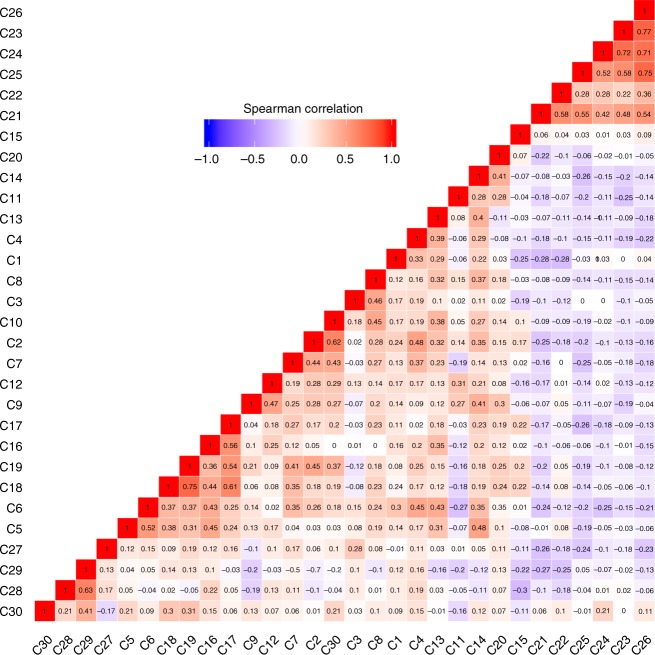
Heatmap with the Spearman’s correlations among the 30 identified and putatively identified metabolites significantly altered. C1: 2-hexanone; C2: hexanal; C3: 2-methylcyclopentan-1-one; C4: 4-methylhexan-3-one; C5: 5-methylheptan-2-one; C6: 4-methyldec-1-ene; C7: 3,7,7-trimethylbicyclo[4.1.0] hept-3-ene; C8: 2,6-dimethyl-6-hepten-2-ol; C9: 3-methyl-6-(propan-2-ylidene)cyclohex-1-ene; C10: 4,6-dimethylheptan-2-one; C11: 3,7-dimethylocta-1,6-dien-3-ol; C12: 3,4-dimethylcyclohex-3-ene-1-carbaldehyde; C13: 1-methyl-4-propan-2-ylcyclohex-2-en-1-ol; C14: terpinen-4-ol; C15: 2,5-dimethylbenzaldehyde; C16: 2-hydroxy-2-methyl-1-phenylpropan-1-one; C17: dihydroedulan IA; C18: 5-methyl-2-(propan-2-yl)cyclohexyl acetate; C19: 2,6,6,10-tetramethyl-1-oxaspiro[4.5]dec-9-ene; C20: 4,5,910-dehydroisolongifolene; C21: 2-butanone; C22: 2-pentanone; C23: cyclohexanone; C24: hexadecane; C25: phenylacetaldehyde; C26: 3-phenylpropionaldehyde; C27: 2-butenal; C28: decanal; C29: glyoxal; C30: methylglyoxal

To overcome the lack of knowledge about the role of volatile compounds in the metabolic pathways, Spearman’s correlation indexes were computed using all identified metabolites (L1 and L2 in Tables 2, 3, S1 and S2) significantly altered in urine of PCa patients (Fig. 3). The magnitude and the sign of correlations can provide identification of metabolites in the same metabolic pathway or under some common regulatory mechanisms. Stronger positive correlations (*r* > 0.7 and *p* < 0.0001) were observed for 2,6,6,10-tetramethyl-1-oxaspiro[4.5]dec-9-ene with 5-methyl-2-(propan-2-yl)cyclohexyl acetate (*r* = 0.75), hexadecane with cyclohexanone (*r* = 0.72), 3-phenylpropionaldehyde with cyclohexanone (*r* = 0.77), 3-phenylpropionaldehyde with hexadecane (*r* = 0.71) and 3-phenylpropionaldehyde with phenylacetaldehyde (*r* = 0.76).

## Discussion

In this study, two HS-SPME/GC-MS approaches were used to more comprehensively uncover the volatile profile of urine from PCa patients compared with previous reports,^8–10^ unveiling a total of 263 different volatile compounds. Multivariate analysis showed that both VOCs and VCCs urinary signature allowed for accurate discrimination between PCa and control groups. A major strength of this study lies in its design, with the inclusion of an external validation set to validate the models obtained through MVA of the training sets, after variable selection. These external validation sets disclosed satisfactory sensitivity (78% for VOCs and VCCs), high specificity (94% for VOCs and 100% for VCCs) and high accuracy (86% for VOCs and 89% for VCCs). Interestingly, all false negatives observed in VOCs model were from obese and/or alcoholic subjects, whereas the false positive was a control with prostatitis (Table 1). Among the four false negatives observed in VCCs model, three were also obese subjects and one with ischaemic heart disease, which may compromise renal function (Table 1). These confounding factors might justify the misclassifications. Notwithstanding, specificity and accuracy were superior to previously published in similar studies.^8,9^ Furthermore, individually, all discriminant metabolites disclosed sensitivity (ranging from 48 to 80%; Tables 2 and 3) higher than the one reported for serum PSA (20.5%).^4^

The idea of using multiple biomarkers rather than a single biomarker has gained strength as a means to improved performance,^25^ since the metabolomic signature of a disease is comprised of groups of connected metabolites that change in concert.^26^ Furthermore, this approach ensures that an arbitrary change in a single metabolite will not lead to a false diagnosis.^26^ In line with this, a biomarker panel was herein defined consisting in the combination of 6 discriminatory metabolites. A small panel of biomarkers was selected in this work envisaging the development of a sensing material^27^ tuned in specificity and selectivity for these compounds to be applied in an “e-nose” in near future. This 6-biomarker panel unveiled good prediction of PCa from non-cancer patients, providing accuracies of 79% and 86% in the internal and external sets, respectively. The small sample size in external set can be considered a limiting factor in this study, though this is the first study, to our knowledge, to use an external set for validation of a volatile biomarker panel of PCa in urine. Importantly, the four patients with BPH and one patient with prostatitis included in the external set as controls were correctly classified by the panel. These prostate non-malignant conditions are well-recognised confounders in the context of serum PSA screening, as elevated levels of this biomarker are detected in BPH and prostatitis.^25^ So, taking into consideration the results of the internal and external validations, the diagnostic performance of the 6-biomarker panel outperforms not only PSA sensitivity but also fPSA/tPSA sensitivity and specificity.

In our study, three classes of compounds stood out as discriminant of PCa from controls, namely alcohols, aldehydes and ketones. A significant decrease was found in the levels of four alcohols, specifically terpinen-4-ol, 2,6-dimethyl-6-hepten-2-ol, 1-methyl-4-propan-2-ylcyclohex-2-en-1-ol, and 3,7-dimethylocta-1,6-dien-3-ol (Table 2). This may be related with changes in several metabolic pathways, namely hydrocarbon metabolism,^28^ fatty acid β-oxidation,^29^ intensification of cellular membrane synthesis^30^ and alterations in the activity of some important enzymes, namely CYP 450^31^ and alcohol dehydrogenases.^28^ Several studies have demonstrated the intracellularly increased concentrations of reactive oxygen species (ROS) in cancer cells,^32,33^ which are capable of causing the oxidation of biologically crucial molecules such as DNA, RNA, proteins and lipids. ROS-mediated oxidation of polyunsaturated fatty acids (also termed lipid peroxidation) increases alkanes formation, which after hydroxylation through CYP 450 leads to the production of alcohols.^31^ Additionally, it has been proposed that terpinen-4-ol and α-terpineol (an isomer of terpinen-4-ol) can interfere with immune response, as they were able to inhibit the production of inflammatory mediators.^34^ Furthermore, α-terpineol was shown to have cytotoxic and apoptotic effects in PCa cell lines, which may be correlated with down-regulation of various proteins that mediate cell proliferation, cell survival, metastasis, and angiogenesis.^35^ 3,7-Dimethylocta-1,6-dien-3-ol may have an exogenous source, since it is present in several food products like cinnamon or citrus fruits.^29^ However, an endogenous origin cannot be ruled out since this compound is involved in lipid metabolism.^29^ In addition, the supplementation with 3,7-dimethylocta-1,6-dien-3-ol in PCa immortalised cell lines and in tumour xenografts showed an induction of apoptosis and inhibition of cell proliferation.^36^

Referring to aldehydes, urinary levels of hexanal, 3,4-dimethylcyclohex-3-ene-1-carbaldehyde, glyoxal, methylglyoxal, decanal, and 2-butenal were found significantly decreased in PCa patients, whereas 2,5-dimethylbenzaldehyde, 3-phenylpropionaldehyde and phenylacetaldehyde were significantly increased in PCa compared to controls (Tables 2 and 3). Aldehydes are involved in the metabolism of alcohols and fatty acids,^37,38^ and can also be produced during amino acid and carbohydrate catabolism.^37,38^ The presence of aldehydes may also be related with the excessive production of ROS,^9^ known to induce lipid peroxidation, which originates the formation of over 200 types of highly reactive and extremely toxic aldehydes.^39^ This may explain the higher levels of 2,5-dimethylbenzaldehyde, 3-phenylpropionaldehyde and phenylacetaldehyde detected in urine of PCa patients. In agreement with our findings, other metabolomic studies have also observed a trend for increased production of certain aldehydes in PCa compared to control groups.^8–10^

The levels of nine ketones were also found significantly altered in urine from PCa patients, including 2-hexanone, 2-methylcyclopentan-1-one, 4-methylhexan-3-one, 5-methylheptan-2-one, 4,6-dimethylheptan-2-one, 2-hydroxy-2-methyl-1-phenyl-propan-1-one, 2-butanone, 2-pentanone and cyclohexanone (Tables 2 and 3). Of note, increased levels of 2-butanone^10^ and decreased 5-methylheptan-2-one levels^40^ were previously associated with PCa in urine samples and cell lines, respectively. Alterations in the levels of ketones might be related with carcinogenic processes, such as protein metabolism and ketogenic pathway dysregulations.^28^ Some important ketones present in the human body are products of fatty acid metabolism, having acetyl-CoA as a precursor.^41^ The increase in ketone levels can also be associated with high oxidation rate of fatty acids and glycation.^42^ During glycation, ROS are formed and contribute to the glycation-induced protein modifications, normally designated glycoxidation.^43^

The exact metabolic pathways which constitute the biological origin of VOCs and VCCs is not completely elucidated yet. Thus far, only one study reported on the cancer-specific biochemical origin of VOCs.^44^ This goal is very difficult to accomplish as VOCs are produced during metabolic cascades as degradation products of the metabolites directly involved in metabolic pathways, and, consequently, conservative methods are unable to determine the VOCs real metabolic origin.^44^ Notwithstanding, some metabolites altered in the PCa group were associated with known biochemical pathways, namely pyruvate metabolism, glycine, serine and threonine metabolism, phenylalanine metabolism and steroid hormone biosynthesis (Fig. S4). However, it is important to take into account that some of the significantly altered metabolites may not be directly cancer-derived but reflect other local or systemic body responses (e.g., inflammation and/or necrosis).

Considering the correlation coefficient (Fig. 3) observed among all identified metabolites (L1 and L2 in Tables 2 and 3 and Tables S1 and S2) found significantly different between cancer and control, the significant decrease in the levels of 2,6,6,10-tetramethyl-1-oxaspiro[4.5]dec-9-ene correlated with the significant decrease in the levels of 5-methyl-2-(propan-2-yl)cyclohexyl acetate, suggesting a possible relationship in PCa disturbed biochemical pathways. Furthermore, we also observed several strong correlations between alterations found in the levels of ketones, aldehydes and alkanes, suggesting a probable association of these compounds with PCa altered metabolism.

Despite the small sample size that may lead to bias in statistical power and precision, our results disclose a volatile biomarker panel that has the potential to be used as a non-invasive diagnostic tool for PCa with good performance. Notwithstanding, the use of a GC-MS approach in routine clinical practice has important limitations, including high cost, non-portability, time-consuming process, and the need for considerable operator expertise.^45^ To overcome these limitations, the use of portable gas-sensing devices such as ‘’e-noses” is a more suitable approach for routine clinical use.^45^ Some research groups have already demonstrated that “e-nose” technology is able to detect the “odour fingerprint” emanated from urine of PCa patients in a simple and fast way.^13,14^

The knowledge on the urinary volatile signature of PCa acquired with this study has the potential to allow for the development of a sensor optimised for the recognition of volatiles with chemical groups herein elucidated and consequently with greater capabilities of chemical discriminations and diagnostic accuracy. However, e-nose devices are incapable to determine the identity and concentration of individual compounds responsible for discrimination between urine samples and, therefore, do not provide information about the metabolic pathways affected by the disease.^46^ Furthermore, the reproducibility of “e-nose” results can be affected by sensor drift over time, affecting instrument reproducibility.^47^ In the future, a best diagnostic approach may rely in the use of low-cost e-nose device for assessing the presence of PCa in a rapid, non-invasive way, followed by targeted assessment of known volatile biomarkers by GC-MS technology for diagnostic confirmation. The combination of e-nose and GC-MS technologies may provide a powerful tandem diagnostic tool potentially allowing for early non-invasive diagnosis of PCa with high accuracy.

## Conclusions

In the present study, a comprehensive volatile metabolomic signature of urine from PCa patients was obtained that covered the profile of a large number of volatile carbonyl compounds reported for the first time. A panel of 6 volatile biomarkers was established for PCa diagnosis, disclosing a good prediction of new PCa and control samples in an external validation cohort. Indeed, the 6-biomarker panel unveiled higher sensitivity and accuracy compared to serum PSA, as well as higher sensitivity and specificity than fPSA/tPSA. The knowledge gained from the definition of PCa volatile signature in urine samples has the potential to be used in the development of an electronic nose device containing sensing materials tuned for specificity and selectivity, thus improving accuracy. Furthermore, the alterations found in the levels of some metabolites (methylglyoxal, phenylacetaldehyde and hexanal) suggest dysregulations in pyruvate metabolism, glycine, serine and threonine metabolism, phenylalanine metabolism and steroid hormone biosynthesis in prostate carcinogenesis. Nonetheless, the biochemical origin of volatile metabolites remains mostly unknown and further studies focused on the understanding of regulatory mechanisms regarding their release at cellular level are required. In conclusion, our findings strengthen the value of urinary volatilome for PCa diagnosis and disclose a biomarker panel that has potential to be used as an accurate diagnostic tool for this malignancy. Further studies will be performed in order to validate these results in an independent larger cohort.

## Supplementary information

Supplementary material_Tables

Supplementary material_Figures

Cover Graphical abstract

## Data Availability

All data that support the findings of this study are included in this published article and its Supplementary information files.
