# Supplementary material for: Identification of a biomarker panel for improvement of prostate cancer diagnosis by volatile metabolic profiling of urine
Source: Br J Cancer. 2019 Oct 7;121(10):857–68. doi: 10.1038/s41416-019-0585-4 (PMC6889512; doi:10.1038/s41416-019-0585-4)
Supplement: Supplementary file 1 — Supplementary material_Tables [file 41416_2019_585_MOESM1_ESM.doc]

**Identification of a biomarker panel for improvement of prostate cancer diagnosis by volatile metabolic profiling of urine**

Ana Rita Lima^1*^, Joana Pinto^1^, Ana Isabel Azevedo^1^, Daniela Barros-Silva^2^, Carmen Jerónimo^2,3^, Rui Henrique^2,3,4^, Maria de Lourdes Bastos^1^, Paula Guedes de Pinho^1*^, Márcia Carvalho^1,5*^

^1^UCIBIO/REQUIMTE, Department of Biological Sciences, Laboratory of Toxicology, Faculty of Pharmacy, University of Porto, Porto, Portugal

^2^Cancer Biology & Epigenetics Group, Research Center (CI-IPOP) Portuguese Oncology Institute of Porto (IPO Porto), Porto, Portugal.

^3^Department of Pathology and Molecular Immunology-Biomedical Sciences Institute (ICBAS), University of Porto, Porto, Portugal.

^4^Department of Pathology, Portuguese Oncology Institute of Porto (IPO Porto), Porto, Portugal.

^5^UFP Energy, Environment and Health Research Unit (FP-ENAS), University Fernando Pessoa, Porto, Portugal.

*Corresponding authors

E-mail addresses:

ritacmlima@hotmail.com (A.R.L.)

pguedes@ff.up.pt (P.G.P.)

mcarv@ufp.edu.pt (M.C.)

Address:

UCIBIO/REQUIMTE, Laboratory of Toxicology

Department of Biological Sciences

Faculty of Pharmacy

University of Porto

Rua Jorge Viterbo Ferreira, 228

4050-313 Porto, Portugal

Tel.: +351 220428599; fax: +351 226093390

Table S1. List of VOCs significantly altered in PCa group compared to controls. They are characterized by their IUPAC name, retention time, characteristic ions (m/z), Kovat indices (KI) from literature, experimental Kovat indices, NIST R-match and CAS registry number.

| **Name** | **Retention time** | **m/z** | **KI from literature** | **Experimental KI or standards** | **R-match** | **CAS number** | **Identification Level^1^** |
| --- | --- | --- | --- | --- | --- | --- | --- |
| **Hexan-2-one**  **(2-Hexanone)** | 4.61 | 58;57;100;85;71 | 790 | 794 | 785 | 591-78-6 | L1 |
| **Hexanal** | 4.797 | 56; 57; 72; 55;99 | 800 | 802 | 823 | 66-25-1 | L1 |
| **2-Methylcyclopentan-1-one** | 5.91 | 98; 55; 69;80 | 847 | 846 | 735 | 1120-72-5 | L1 |
| **4-Methylhexan-3-one** | 6.86 | 57; 85; 72; 58; 114;55 | 853 | 884 | 920 | 17042-16-9 | L2 |
| **Unknown 1** | 7.92 | 59; 56; 55; 76; 84; 65; 57 | - | 921 | - | - | L4 |
| **5-Methylheptan-2-one** | 9.22 | 58; 71; 70; 55;57;56;59;87;74 | 971 | 966 | 659 | 18217-12-4 | L1 |
| **4-Methyldec-1-ene** | 10.00 | 57; 56; 41;71;112;55 | 1041 | 992 | 685 | 13151-29-6 | L2 |
| **3,7,7-Trimethylbicyclo[4.1.0] hept-3-ene (3-Carene)** | 10.40 | 93;79;77;92;121;80; 94;105; 136; 53 | 1011 | 1006 | 907 | 13466-78-9 | L1 |
| **2,6-Dimethyl-6-hepten-2-ol** | 10.47 | 59;69;56;109;68;124 | 996 | 1008 | 650 | 32779-58-1 | L1 |
| **3-Methyl-6-(propan-2-ylidene)cyclohex-1-ene (Isoterpinolene)** | 10.68 | 121; 93; 136; 79; 91; 77;105;107;67; 53 | 1023 | 1015 | 842 | 586-63-0 | L2 |
| **4,6-Dimethylheptan-2-one** | 11.82 | 58;85;69;84;59;57;53 | 1045 | 1052 | 802 | 19549-80-5 | L2 |
| **3,7-Dimethylocta-1,6-dien-3-ol** **(Linalool)** | 13.19 | 71; 93; 69; 55; 80; 121; 67 | 1082 | 1098 | 708 | 78-70-6 | L1 |
| **Unknown 2** | 13.73 | 119; 108; 99; 107; 70; 120; 111; 139 | - | 1116 | - | - | L4 |
| **Unknown 3** | 13.84 | 79; 91; 94; 109;119;77;121;81 | - | 1120 | - | - | L4 |
| **3,4-Dimethylcyclohex-3-ene-1-carbaldehyde** | 14.11 | 67;138;95;91;79;93;105 | 1130 | 1129 | 805 | 18022-66-7 | L1 |
| **1-Methyl-4-propan-2-ylcyclohex-2-en-1-ol** | 15.09 | 93;139;69;121;111;55;71; 81 | 1122 | 1162 | 675 | 29803-81-4 | L2 |
| **Unknown 4** | 15.74 | 100; 55; 81; 70; 69; 128; 56; 67 | - | 1184 | - | - | L4 |
| **4-Methyl-1-propan-2-ylcyclohex-3-en-1-ol (Terpinen-4-ol)** | 16.00 | 59; 93; 121; 136; 67;81;68;79;91 | 1189 | 1193 | 904 | 98-55-5 | L1 |
| **Unknown 5** | 16.26 | 135; 55; 91; 164; 136; 79; 53; 65; 77; 105 | - | 1202 | - | - | L4 |
| **Unknown 6** | 16.35 | 61;89;143;81;75;137; 135;115;55;67 | - | 1205 | - | - | L4 |
| **2,5-Dimethylbenzaldehyde** | 16.50 | 133; 134; 105; 91; 77 | 1208 | 1176 | 841 | 5779-94-2 | L1 |
| **2-Hydroxy-2-methyl-1-phenylpropan-1-one** | 18.45 | 59;77;105;50;106;51; 78 | 1278 | 1277 | 808 | 7473-98-5 | L1 |
| **2,5,5,8-Tetramethyl-3,4,4,6-tetrahydro-2-H-chromene** | 18.60 | 179;107;84;55;95;91;77;69 | 1293 | 1287 | 600 | 41678-32-4 | L2 |
| **5-Methyl-2-(propan-2-yl) cyclohexyl acetate**  **(Menthyl acetate)** | 18.80 | 95; 138; 81; 123; 96; 82; 94; 67 | 1304 | 1296 | 709 | 89-48-5 | L1 |
| **2,6,6,10-Tetramethyl-1-oxaspiro[4.5]dec-9-ene (Theaspirane)** | 19.40 | 138; 82; 96; 83; 139; 109; 55; 123 | 1302 | 1312 | 837 | 36431-72-8 | L1 |
| **Unknown 7** | 20.53 | 56; 67; 79; 83;147; 53;114; 89; 98; 52 | - | 1358 | - | - | L4 |
| **Unknown 8** | 21.43 | 135; 73; 79; 70; 91; 77; 133; 55; 123; 67 | - | 1392 | - | - | L4 |
| **Unknown 9** | 21.83 | 91; 67; 53; 79;77 | - | 1408 | - | - | L4 |
| **Unknown 10** | 24.68 | 69; 105; 129; 59; 93; 68; 67; 77 | - | 1522 | - | - | L4 |
| **Unknown 11** | 24.74 | 57; 55; 191; 113; 70; 56; 117; 69 | - | 1525 | - | - | L4 |
| **2,2,7,7-Tetramethyltricyclo[6.2.1.0¹,⁶]undeca-3,5,9-triene (4,5,9,10-dehydroisolongifolene)** | 25.22 | 107;159;205;131;91;117;220;163;105;187 | 1544 | 1546 | 678 | NA | L2 |

^1^: Viant MR, Kurland IJ, Jones MR and Dunn WB (2017) How close are we to complete annotation of metabolomes? Curr Opin Chem Biol 36:64-69. L1: Identified metabolites (GC-MS analysis of the metabolite of interest and a chemical reference standard of suspected structural equivalence, with all analyses performed under identical analytical conditions within the same laboratory); L2: Putatively annotated compounds (spectral (MS) similarity with NIST database), when standards were not commercially available; L4: Unidentified.

Table S2. List of VCCs significantly altered in PCa group compared to controls. They are characterized by their IUPAC name, retention time, characteristic ions, Kovat indices (KI) from literature, experimental Kovat indices, NIST R-match and Cas registry number.

| **Name** | **Retention time** | **m/z** | **KI from literature** | **Experimental KI or standards** | **R-match** | **CAS number** | **Identification Level** |
| --- | --- | --- | --- | --- | --- | --- | --- |
| **Unknown 12** | 16.19 | 163;207;164;143;113 | - | 1194 | - | - | L4 |
| **Butan-2-one**  **(2-Butanone)** | 17.35;17.43 | 56;250;86;195;267 | 1335 | 1235/1238 | 838;844 | 78-93-3 | L1 |
| **Pentan-2-one**  **(2-Pentanone)** | 19.31 | 253;42;41;56;72;100;182 | 1434 | 1305 | 868 | 107-87-9 | L1 |
| **But-2-enal**  **(2-Butenal)** | 20.09 | 250;182;161;195;117 265 | 1339 | 1335 | 721 | 123-73-9 | L1 |
| **Cyclohexanone** | 24.91 | 82;67;293;81;112;276;  54;55 | 1635 | 1549 | 759 | 108-94-1 | L1 |
| **Hexadecane** | 25.69 | 72;57;71;85;55;56 | 1600 | 1613 | 836 | 544-76-3 | L1 |
| **Unknown 13** | 28.20 | 95;107;75;83;108;319;  161;182;289 | - | 1765 | - | - | L4 |
| **Phenylacetaldehyde** | 29.44 | 91,117, 65; 182, 315, 297, 77, 161, 134 | 1832 | 1919 | 755 | 122-78-1 | L1 |
| **3-Phenylpropanal**  **(3-Phenylpropionaldehyde)** | 30.67 | 91;104;105;117;77;103;  65;271;130 | 1931 | 1980 | 703 | 104-53-0 | L1 |
| **Decanal** | 31.80 | 239;170;182;55;57;69;  240 | 1954 | 2130 | 825 | 112-31-2 | L1 |
| **Oxaldehyde**  **(Glyoxal)** | 33.54 | 182;161;195;167;117;99;448;93;119 | 1935 | 2221 | 933 | 107-22-2 | L1 |
| **2-Oxopropanal**  **(Methylglyoxal/ Pyruvaldehyde)** | 34.30 | 182;265;161;195;167;  117;99;119;168 | 2174 | 2260 | 935 | 78-98-8 | L1 |

^1^: Viant MR, Kurland IJ, Jones MR and Dunn WB (2017) How close are we to complete annotation of metabolomes? Curr Opin Chem Biol 36:64-69. L1: Identified metabolites (GC-MS analysis of the metabolite of interest and a chemical reference standard of suspected structural equivalence, with all analyses performed under identical analytical conditions within the same laboratory); L2: Putatively annotated compounds (spectral (MS) similarity with NIST database); L4: Unidentified.

**Table S3.** Confusion matrix obtained for VOCs and VCCs considering the external validation sets (*n*=18 PCa patients plus *n*=18 cancer-free controls).

| True Classes |  | Predicted Classes | | | | |
| --- | --- | --- | --- | --- | --- | --- |
|  |  | VOCs | |  | VCCs | |
|  |  | *Case* | *Control* |  | *Case* | *Control* |
|  | *Case* | 14 | 4 |  | 14 | 4 |
|  | *Control* | 1 | 17 |  | 0 | 18 |

**Table S4.** Spearman’s correlation indexes and corresponding *p*-values obtained for age with the set of metabolites found altered in PCa compared to controls.

| **Metabolites** | ***r*** | ***p*** |
| --- | --- | --- |
| ***VOCs*** |  |  |
| 2-Hexanone | -0.08 | 0.4578 |
| Hexanal | 0.00 | 0.9670 |
| 2-Methylcyclopentan-1-one | -0.10 | 0.3915 |
| 4-Methylhexan-3-one | -0.01 | 0.9114 |
| Unknown 1 | -0.15 | 0.1860 |
| 5-Methylheptan-2-one | -0.22 | 0.0514 |
| 4-Methyldec-1-ene | -0.15 | 0.1674 |
| 3,7,7-Trimethylbicyclo[4.1.0] hept-3-ene (3-Carene) | 0.00 | 0.9764 |
| 2,6-Dimethyl-6-hepten-2-ol | -0.29 | 0.0082 |
| 3-Methyl-6-(propan-2-ylidene)cyclohex-1-ene (Isoterpinolene) | -0.22 | 0.0483 |
| 4,6-Dimethylheptan-2-one | -0.25 | 0.0249 |
| 3,7-Dimethyl-1,6-octadien-3-ol (Linalool) | -0.23 | 0.0358 |
| Unknown 2 | -0.28 | 0.0115 |
| Unknown 3 | -0.32 | 0.0038 |
| 3,4-Dimethylcyclohex-3-ene-1-carbaldehyde | -0.31 | 0.0041 |
| 1-Methyl-4-propan-2-ylcyclohex-2-en-1-ol | -0.30 | 0.0071 |
| Unknown 4 | -0.39 | 0.0004 |
| 4-Methyl-1-propan-2-ylcyclohex-3-en-1-ol (Terpinen-4-ol) | -0.36 | 0.0010 |
| Unknown 5 | -0.19 | 0.0814 |
| Unknown 6 | 0.20 | 0.0742 |
| 2,5-Dimethylbenzaldehyde | 0.19 | 0.0929 |
| 2-Hydroxy-2-methyl-1-phenylpropan-1-one | -0.23 | 0.0368 |
| 2,5,5,8-Tetramethyl-3,4,4,6-tetrahydro-2-H-chromene | -0.14 | 0.2157 |
| 5-Methyl-2-(propan-2-yl) cyclohexyl acetate (Menthyl acetate) | -0.16 | 0.1505 |
| 2,6,6,10-Tetramethyl-1-oxaspiro[4.5]dec-9-ene (Theaspirane) | -0.13 | 0.2397 |
| Unknown 7 | -0.09 | 0.4011 |
| Unknown 8 | -0.20 | 0.0692 |
| Unknown 9 | -0.16 | 0.1543 |
| Unknown 10 | -0.24 | 0.0333 |
| Unknown 11 | -0.05 | 0.6494 |
| 2,2,7,7-Tetramethyltricyclo[6.2.1.0¹,⁶]undeca-3,5,9-triene (4,5,9,10-dehydroisolongifolene) | -0.19 | 0.0889 |
| ***VCCs*** |  |  |
| Unknown 12 | 0.12 | 0.2991 |
| 2-Butanone | 0.04 | 0.7035 |
| 2-Pentanone | 0.02 | 0.8925 |
| 2-Butenal | -0.02 | 0.8418 |
| Cyclohexanone | 0.03 | 0.7650 |
| Hexadecane | 0.04 | 0.7433 |
| Unknown 13 | -0.01 | 0.9429 |
| Phenylacetaldehyde | 0.20 | 0.0705 |
| 3-Phenylpropionaldehyde | 0.09 | 0.4186 |
| Decanal | -0.17 | 0.1341 |
| Oxaldehyde (Glyoxal) | -0.08 | 0.5009 |
| 2-Oxopropanal (Methylglyoxal/Pyruvaldehyde) | -0.07 | 0.5409 |

**Table S5.** Confusion matrix obtained for the panel of 6 metabolites considering the external validation set (*n*=18 PCa plus *n*=18 control) (sensitivity: 88%, specificity: 83% and accuracy: 86%).

| True Classes |  | Predicted classes | | |
| --- | --- | --- | --- | --- |
|  |  | | *Case* | *Control* |
|  | *Case* | | 16 | 2 |
|  | *Control* | | 3 | 15 |
