# Supplementary material for: Identification of a biomarker panel for improvement of prostate cancer diagnosis by volatile metabolic profiling of urine
Source: Br J Cancer. 2019 Oct 7;121(10):857–68. doi: 10.1038/s41416-019-0585-4 (PMC6889512; doi:10.1038/s41416-019-0585-4)
Supplement: Supplementary file 2 — Supplementary material_Figures [file 41416_2019_585_MOESM2_ESM.docx]

**Identification of a biomarker panel for improvement of prostate cancer diagnosis by volatile metabolic profiling of urine**

Ana Rita Lima^1*^, Joana Pinto^1^, Ana Isabel Azevedo^1^, Daniela Barros-Silva^2^, Carmen Jerónimo^2,3^, Rui Henrique^2,3,4^, Maria de Lourdes Bastos^1^, Paula Guedes de Pinho^1*^, Márcia Carvalho^1,5*^

^1^UCIBIO/REQUIMTE, Department of Biological Sciences, Laboratory of Toxicology, Faculty of Pharmacy, University of Porto, Porto, Portugal

^2^Cancer Biology & Epigenetics Group, Research Center (CI-IPOP) Portuguese Oncology Institute of Porto (IPO Porto), Porto, Portugal.

^3^Department of Pathology and Molecular Immunology-Biomedical Sciences Institute (ICBAS), University of Porto, Porto, Portugal.

^4^Department of Pathology, Portuguese Oncology Institute of Porto (IPO Porto), Porto, Portugal.

^5^UFP Energy, Environment and Health Research Unit (FP-ENAS), University Fernando Pessoa, Porto, Portugal.

*Corresponding authors

E-mail addresses:

ritacmlima@hotmail.com (A.R.L.)

pguedes@ff.up.pt (P.G.P.)

mcarv@ufp.edu.pt (M.C.)

Address:

UCIBIO/REQUIMTE, Laboratory of Toxicology

Department of Biological Sciences

Faculty of Pharmacy

University of Porto

Rua Jorge Viterbo Ferreira, 228

4050-313 Porto, Portugal

Tel.: +351 220428599; fax: +351 226093390


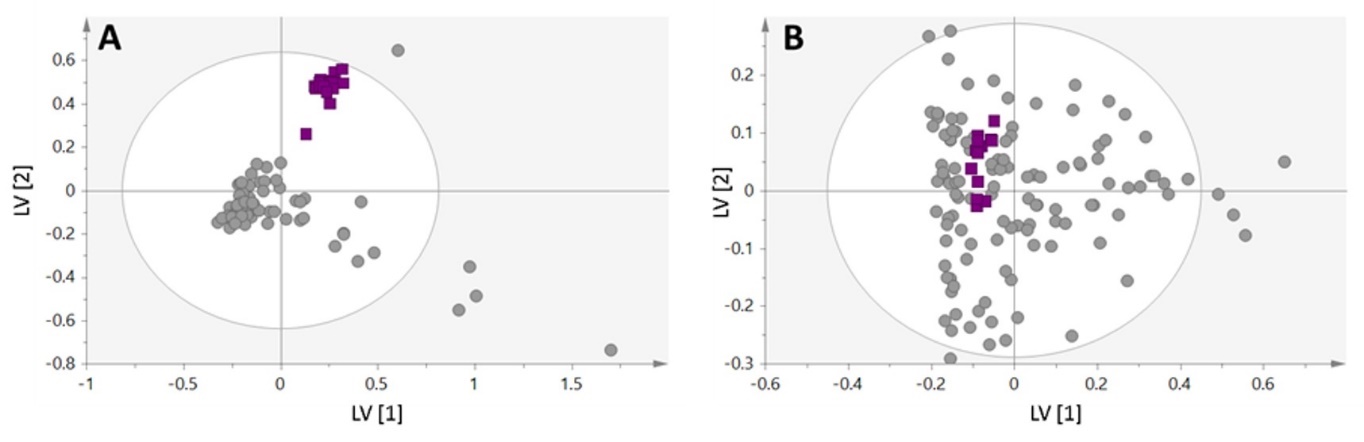


**Figure S1.** PCA scores scatter plot (Pareto scaling; 2 components) obtained for the HS-SPME/GC-MS chromatograms of all samples (control and PCa) (gray circles) and QCs samples (purple squares). (A) VOCs (R^2^X=0.345); (B) VCCs (R^2^X=0.423). QCs samples are grouped together, which prove the reproducibility of the analytical techniques.


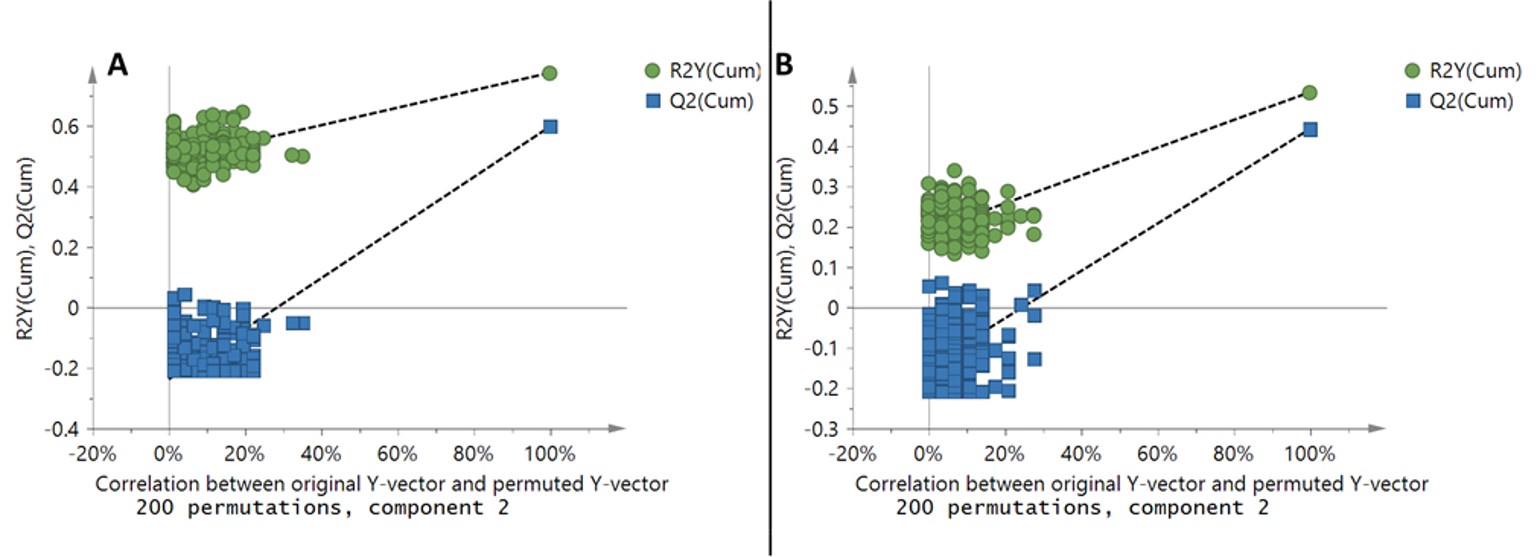


**Figure S2.** Statistical validation of the PLS-DA models by permutation testing (200 permutations; 2 components). (A) VOCs model (Intercepts: R^2^= (0.0, 0.487), Q^2^= (0.0, -0.237)). (B) VCCs model (Intercepts: R^2^= (0.0, 0.189), Q^2^= (0.0, -0.144)).


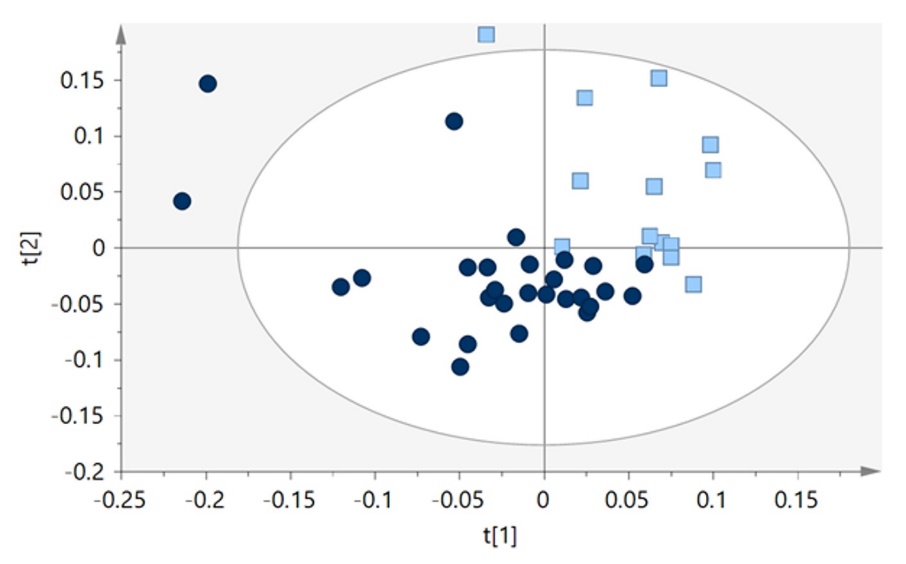


**Figure S3.** PLS-DA scores scatter plots (Pareto scaling; 2 components) obtained for control without hypertension (dark blue circles) vs. control with hypertension (light blue squares) (LV= 2, R^2^X= 0.193; R^2^Y= 0.617; Q^2^= -0.145) (n= 28 controls without hypertension vs n= 14 controls with hypertension).


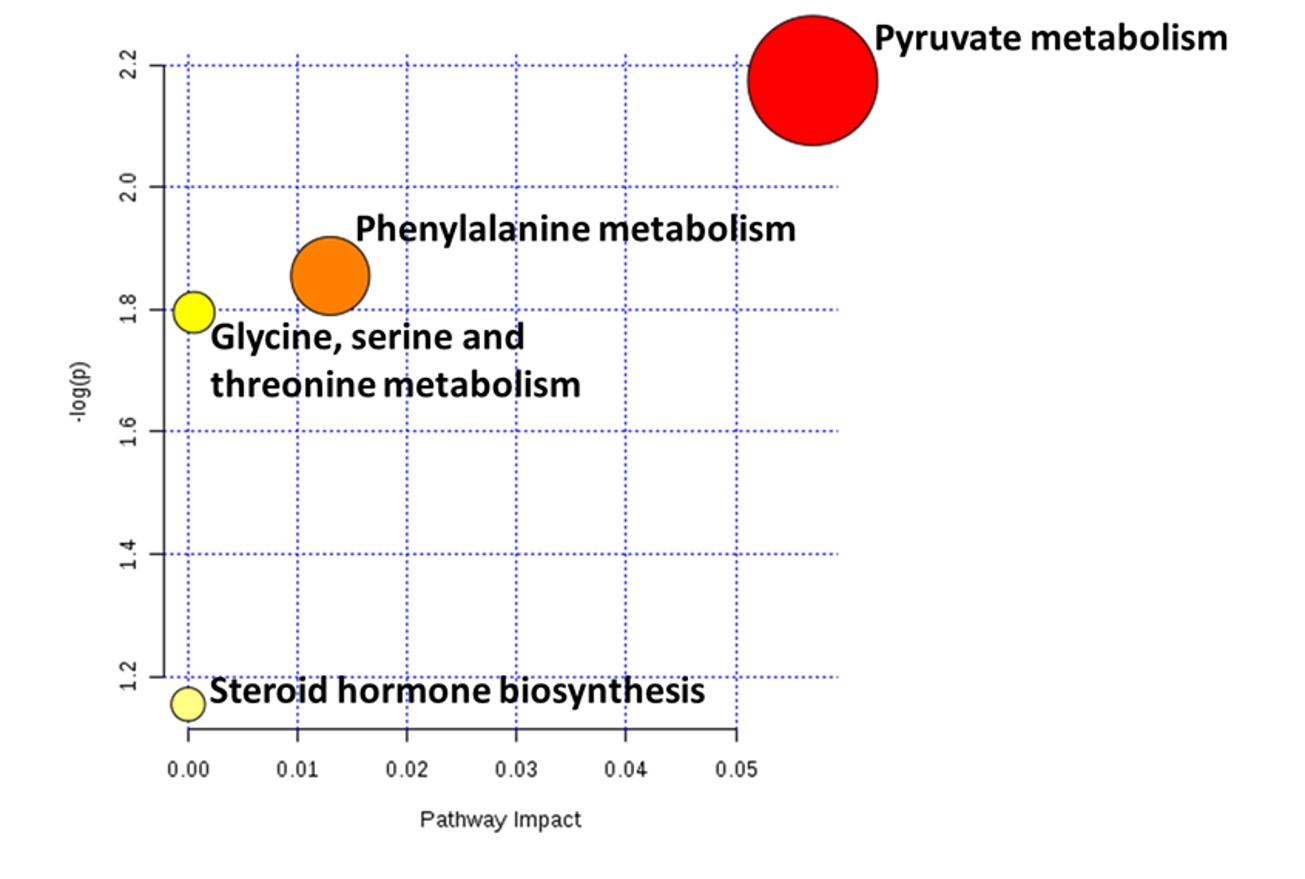


**Figure S4.** Metabolic pathway analysis performed by the MetPA tool in Metaboanalyst 3.0. Pathway topology analysis depicting dysregulated metabolic pathways in PCa patients. The X-axis represents the pathway impact values, and the Y-axis indicates the -log of p-values from the pathway enrichment analysis. The color of the nodes links to the p-values and the node radius is linked to the pathway impact values.
