# Supplementary material for: Identification of a biomarker panel for improvement of prostate cancer diagnosis by volatile metabolic profiling of urine
Source: Br J Cancer. 2019 Oct 7;121(10):857–68. doi: 10.1038/s41416-019-0585-4 (PMC6889512; doi:10.1038/s41416-019-0585-4)
Supplement: Supplementary file 3 — Cover Graphical abstract [file 41416_2019_585_MOESM3_ESM.docx]

A HS-SPME/GC-MS based metabolomics approach was performed to evaluate the performance of VOCs and VCCs excreted in urine to discriminate PCa patients from non-cancer subjects. Considering the obtained profiles, a panel of 6 volatile compounds able to discriminate PCa was defined, which proved a high performance (sensitivity of 89%, specificity of 83% and accuracy of 86%) to predict new PCa and control samples in an external validation set.
